# Revealing gendered identity and agency in dementia

**DOI:** 10.1111/hsc.12452

**Published:** 2017-05-03

**Authors:** Geraldine Boyle

**Affiliations:** ^1^ School of Health, Wellbeing and Social Care The Open University Milton Keynes UK

**Keywords:** agency, dementia, dialogue, gender, identity, well‐being

## Abstract

As identity and agency are central to the well‐being of people with dementia, this paper explores whether their dialogue conveys a gendered sense of identity and agency. The author discusses whether they demonstrate not just a subjective sense of being but also an understanding of their relational selves. Findings are presented from a qualitative study in the North of England which examined the everyday decisions made by married couples when one partner had dementia. Ethnographic methods were used, including participant observation and interviews. While dialogical analysis usually centres on the subjective self, it was also used to examine intersubjectivity. Comparisons are made between the dialogue of women and men in order to draw conclusions about the gendered nature of identity and agency. The study found that the women and men defined themselves according to their social and gender identities. The literature had suggested that agency might be a gendered concept and the study confirmed that men were somewhat individualistic and rational in their concerns, whereas women were more relational and even spiritual. Yet, women and men demonstrated emotional reflexivity. As national and international health policy prioritises living well with dementia, more systematic attention should be given to the role of gender in influencing well‐being in dementia. Health and social care staff should recognise and facilitate the gender identity and related social roles of people with dementia (e.g. parent, carer and worker) in order to enhance their quality of life.

**What is known about this topic**

- Identity and agency are central to the well‐being of people with dementia.
- Previous research has shown that people with dementia can convey gender identity in material or embodied forms.

**What this paper adds**

- Analysis of the dialogue of people with dementia shows that they define themselves according to their social and gender identities.
- People with dementia convey gendered expressions of agency to some extent, but women *and* men demonstrate emotional reflexivity.

## 1 Introduction

The global population living with dementia will double to 65.7 million by 2030. Accordingly, the World Health Organization (2012) has designated dementia as a public health priority. In the UK, the Prime Minister's Challenge 2020 aims to support living well with dementia (Department of Health, 2016). As identity and agency are central to the well‐being of people with dementia, this paper explores what their dialogue conveys about their sense of identity and agency (Kitwood & Bredin, 1992). As the World Health Organization (2012) did not consider the role of gender in influencing well‐being in dementia—referring only to sex differences in prevalence rates—the author discusses what their dialogue reveals about *gendered* identity and agency.

The concept of dialogue relates to an engagement with the self and a dialogue between self and others (Sullivan & McCarthy, 2004). One's voice is “permeated with the voices of others”—some are embraced while others are contested or resisted (Frank, 2005; p. 968). As these voices are often implicit and even unconscious, the dialogue may be implied or indirect (Burkitt, 2010). Although dialogical analysis centres on subjectivity, this approach seems suited to examining intersubjectivity, particularly as a focus on self‐identity in dementia research has led to the latter being neglected (Caddell & Clare, 2010; Sullivan, 2012). I discuss whether people with dementia demonstrate not just a subjective sense of being but also an understanding of their social selves, rooted in families and communities.

Research into the experience of dementia has lacked a gender perspective (Alzheimer's Disease International, 2015). A study of dress in dementia showed how material culture such as handbags can be used to express gender identity when living with the condition (Buse & Twigg, 2014). Other research has highlighted how their grooming practices remain gendered (Ward, Campbell, & Keady, 2014). However, these studies focused on material or embodied representations of gender rather than how this is shaped through dialogue (Davis, 1991). Borley and Hardy (2016) investigated the transition from being a carer to receiving care in dementia from a woman's perspective, but their study did not consider gender beyond caring identities. Thus, a broader exploration of identity was needed, particularly whether participants defined themselves in terms of dementia or gender (or both), either explicitly or implicitly.

Theoretical perspectives on gender have shifted significantly from biological to sociological explanations (Chakravarti, 2015). The biological standpoint innately linked “sex” with personality traits and roles—notably masculine independence, mastery and the provider role or feminine relationality, intimacy and a caring role (Matud, Ibanez, & Bethencourt, 2003). Men were portrayed as rational, self‐reliant and powerful, in contrast to women's assumed emotionality, dependency and passivity (Kvigne et al., 2014; Suh et al., 2004). On the contrary, however, gender is socially constructed (Davis, 1991). While our gendered self is bounded by societal norms, it is also performed and redefined in everyday relations (West & Zimmerman, 1987).

Previous research has often disregarded whether people with dementia can exercise agency, although studies have explored how self‐help groups can promote their empowerment (Orulv, 2012). Yet, agency is the means by which the subjective self becomes a social self (Burkitt, 2008). While Giddens (1991) conceptualised reflexive agency as an individualistic reasoning process, on the contrary, it is relational, dialogical and emotionally driven (Burkitt, 2012). Thus, agency involves engaging socio‐emotionally with the lives and concerns of ourselves and others (Boyle, 2015). There is also a lack of research into how dialogue informs on agency, despite the key relevance of the latter concept to gender and health debate. Gomersall, Madill, and Summers (2012) used dialogical analysis to explore women's accounts of self‐managing diabetes, but their study did not explicitly examine agency. However, Townsend et al. (2014) used narrative analysis to examine help‐seeking among women with rheumatoid arthritis and found that they exhibited some masculine behaviours, such as delaying GP consultations in order to retain personal control over the illness. Yet, neither study directly compared the gendered constructions of women and men. In addition, such studies narrowly focus on agency manifested in illness management or help‐seeking rather than the individual's fundamental agency, *beyond* the illness. Thus, whether agency takes gendered forms has not been adequately considered.

This paper draws on findings from a study of everyday decision‐making by people with dementia and their spouses which explored the role of social factors in influencing their decisions. I discuss whether women and men with dementia expressed their own voices—and echoed or anticipated the voices of others—and what these conveyed about gender identity and agency.

## 2 Methods

An intensive, qualitative methodology was used to examine decision‐making. Although the overall study included people with advanced dementia, this paper focuses on the participants with mild or moderate dementia who usually had more discursive ability. The research was undertaken within a metropolitan local authority in the North of England. Ethics approval was obtained from the national Social Care Research Ethics Committee.

Ethnographic methods were used, including participant observation and interviews. The fieldwork was undertaken over at least four home visits and each couple was observed going about their daily routines (e.g. preparing a meal), in order to observe decision‐making directly. While semi‐structured interviews were undertaken with each spouse, this paper concentrates on the interviews with people with dementia, in order to explore their thoughts and feelings in depth. A life course perspective located them within their personal and cultural biographies, for example, previous occupations and family roles (Hendricks, 2011). Photographs of the couples (e.g. undertaking social activities) were used to prompt recall (Harper, 2002). Their non‐verbal communication (e.g. facial expressions) was observed, in order to enhance understanding (Mayhew et al., 2001).

The couples were recruited via Wellbeing Cafes and support groups for people with dementia and their carers. Theoretical sampling was used to recruit women and men with dementia and ensure diversity in their cognitive and communication abilities (e.g. by recruiting participants with limited speech who used non‐verbal communication). The couples were eligible to participate if they were co‐resident; one spouse had been diagnosed with dementia and both spouses were willing to take part. Written consent was obtained; alternatively, people with dementia who had writing difficulties gave verbal consent. The fieldwork was completed in 2011. The interviews were digitally recorded and transcribed verbatim.

As the interviews did not adopt an explicitly gendered approach, the data analysis explored whether the participants perceived and represented themselves and their involvement in social relationships in gendered terms—such as characteristics, abilities and roles—and how they negotiated others' gendered assumptions. In line with dialogical theory, the analysis illustrates their “struggles of becoming” (Frank, 2005, p. 968). Their deliberative or executional agency was highlighted and also instances which demonstrated their socio‐emotional (including embodied) reflexivity. As per the dialogical approach, the textual analysis focused on the “emotionally laden stories” they revealed, that is, anecdotes conveyed with particular emotional expression (Gomersall et al., 2012). Likewise, the audio‐recordings were carefully listened to for evidence of speech patterns and tone of voice which revealed the emotive nature of their stories. Understanding the participants' implicit meanings required knowledge of their personal and sociocultural contexts (e.g. marriage, family, religion, ethnicity), additionally informed by the observation, photo‐elicitation and interviews with spouses. To ensure the validity of our interpretations, these were often checked at the time of the fieldwork.

This paper focuses on eight women and eight men with dementia who had the capacity to consent to taking part in the research (as per the Mental Capacity Act^2005^ in England and Wales). Each of this subgroup had mild or moderate dementia and a few had limited speech. The duration since diagnosis varied from 1 to 6 years. Their ages ranged from 50s to 80s. The subgroup was predominantly white British, but included one British Pakistani man. As this was a small sample, the findings may not be generalisable to women and men with dementia more broadly.

## 3 Findings

### 3.1 What matters for women's and men's identity and agency?

#### 3.1.1 Relationships matter

The significance of intimate relationships was the primary theme mentioned by all the women—usually the couple's relationship but also family relationships. Although men prioritised work, most of them also identified the importance of these relationships in their lives (six out of eight).

#### 3.1.2 Sustaining relationships

When women were asked about themselves, they often referred instead to the couple, suggesting that they prioritised the couple identity. Amanda^1^ said the couple were close: “we're joined at the hip”. She demonstrated emotional reflexivity when she said her love for her husband waxed and waned, reflecting the challenges of maintaining emotional intimacy, irrespective of dementia: “when I love him, I love him; when I don't love him, I like him and when I don't like him, I love him”. At times, the references made to a couple were explained by shared social lives, often intensified by the onset of dementia (Molyneaux et al., 2011). Derek and his wife spent a lot of time together now, but their social lives had been quite separate in their earlier marriage. The emphasis on the couple identity may have helped some women to counter a faltering sense of self (see Williams, 1987). Paula contrasted her husband's competency with her own forgetfulness, suggesting she felt lacking in competency: “he's competent, he can remember things which I would be forgetting”.

Only one man with dementia emphasised a strong sense of jointness within the couple (although others referred to their shared lives), suggesting that men gave more priority to their individual identity. Alfred said his marriage had been the most important aspect of his life: “(I) met Susan, that was the biggest part of my life was that. It still is”. His marriage seemed to have brought him emotional stability and love that had been relatively absent from his childhood: “an aunt took me … my mother just, er, only thought of herself, really. So, I didn't really get much, much love in the home life”. As Alfred's personal identity was dominated by a sense of being unwanted as a child, the couple identity seemed to provide him with an affirmative substitute.

Five participants (including two men) highlighted the importance of their children or grandchildren in their lives. Patricia described the close bond between her and her daughter in quite a pedantic way, laughing to show her awareness that what she was saying was self‐evident: “well, we are close, 'cause I'm her mother” (she had limited speech). She perused a family album during her interview, noticeably laughing at photos of her daughter playing as a child. Paul referred several times to his daughter and said the couple could rely on her for support. Their descriptions were somewhat gendered, as he referred to his parental relationship in terms of receiving practical support, whereas Patricia emphasised their emotional bond, despite her greater need for disability support. Although she had impaired speech, her capacity for emotional expression enabled her to sustain a cherished relationship and, in turn, her maternal identity.

#### 3.1.3 Gendered tensions within relationships

At times, the dialogue pointed to gendered tensions within a couple. Three women with dementia bemoaned the pastimes they shared with their husbands—notably masculine sports—when they would have preferred to engage in other social activities. Carol accompanied her husband to cricket matches because William did not want to leave her on her own and she acquiesced to keep him happy. Yet, she often did not want to go to the matches—“well, sometimes I just don't want to go”—and would have preferred to attend a Wellbeing Café instead (which might have been more beneficial for her well‐being). Amanda said the couple regularly attended a sports event when she would have preferred to go to church, but her husband disapproved of her religious practice: “yes, but don't tell him that”. The husband‐carers' rationale for expecting their wives to accompany them was that they disliked leaving them alone now that they had dementia, but wife‐carers did not necessarily expect their husbands to join them in female‐oriented pastimes. As these women had less opportunity to focus on their personal interests, there was also less scope for deliberative agency.

As regards tensions within two couples over domestic roles, Paula said she would rather pursue a hobby than do housework: “I'm not going to sweep the floor every day, um … I'd rather be playing the piano”. Yet, despite her dislike of domestic chores, these remained her responsibility. Carol did not attach much importance to housekeeping either but, again, this was her remit: “(it) gets done when it gets done”. As she also mentioned a few times in her interview that her husband would not let her cook, it appeared that she was unhappy about this. She did not discuss this domestic division of labour with William, preferring to avoid friction: “I keep my mouth shut”. As her husband excluded her from cooking because of her condition, this mirrors welfare concerns about domestic competency in dementia (Marshall, 2004).

Sid seemed ambiguous about his marriage, but as he joked a lot it was not clear if he was being serious. Initially, he said he would not get married again—“I wouldn't do it again, let's put it that way”—but then said “you know, there's nowt wrong with it” and that his wife, Rose, was “a good 'un”. Yet, Rose revealed in her interview that he had been domineering and “quite violent” earlier in their marriage. Although she had to take the lead in decision‐making now—including financial decisions—it appeared that Sid had difficulty relinquishing deliberative agency here, particularly as Rose said he had been quite controlling about money in their earlier marriage: “he's lost that bit of control and he don't like it”. He acknowledged that Rose made some decisions for him: “she comes out and asks me what we're gonna do, and I'll say ‘I don't know’”. Mr. Pasha had been used to exercising decision‐making authority throughout his marriage but his wife had to take the lead in decisions now. Yet, he appeared to be resisting this shift in decision‐making control. He acknowledged that his wife made a lot of the decisions and that he frequently disagreed with her, but he minimised the disagreement which arose: “I rely 100%, you know, on her because she's the one after the, you know, who takes charge … Her decision, sometime I don't like it and I disagree and then things do go ahead, you know? Whether I disagree or agree, right? … you know, it's only a bit, a little dispute, a dispute that's all”. Mrs. Pasha revealed in her interview (in Urdu, via an interpreter) that her husband had become physically aggressive. Aggression can occur in people with dementia, but may also precede its onset (Mowlam et al., 2007). The men's perspectives show that exercising decision‐making authority within marital relationships is important for masculinity even in dementia (see Connell, 1995).

### 3.2 Men at work

The importance of paid work was the key theme mentioned by six men with dementia, suggesting that occupation—or instrumental agency—was central to their masculinity (irrespective of social class). Paul described his engineering job in detail, conveying pride in his technical competency: “we designed the jigs and the tools, and for, for manufacturing the pistons”. Indeed, he seemed most comfortable with his sense of self when describing his occupation. Martin was quite explicit that the loss of his livelihood challenged his manhood: “I'm not the man I was. And I hate it … because I've, I've never been out of work”.

Some men had had to give up their jobs prematurely, whether because of the dementia or other health problems. Mr. Pasha had to give up work in his early 40s due to physical ill‐health: “I was broken‐hearted … after the heart attack, there was no chance of me working because I was used to catering business and I couldn't find any other job that suit me”. Martin sighed as he recounted the episode when his employer had received a letter from his doctor regarding his Alzheimer's (although he himself had not yet been informed of the diagnosis) and his career was immediately terminated. He said the woman who did the “hiring and firing” had declared “you're finishing there”, which made him feel “a bit awful. I've, I've never been sacked”. His emotive narrative conveyed how abruptly he had been removed from his post and that he had clearly found this distressing, illustrating his emotional reflexivity.

The men showed how their occupational personas remained strong. Gordon often spoke as if he was still employed as a health professional and, accordingly, sought to take charge. For example, he viewed himself as overseeing the day service he attended: “I was full of, um, enthusiasm for it because it's a chance to raise the level of the expectations for the, the department”. Peter's enduring work identity was conveyed embodily as he had impaired speech. His wife said (in her interview) that when he attended the day centre, he still dressed for the workplace.

Most of these men said they would still prefer to be employed. Sid said: “I'd love to go back to work”. Mr. Pasha attributed his depression to his loss of a working life, particularly as he had been the sole breadwinner for his family and this status was important in cultural terms and, in turn, informed his masculinity (as a British/Pakistani Muslim man): “I do miss the work because, erm, staying home all the time for some time now and, er, it's put to (caused) depression”. In identifying work as crucial to their masculinity, men with dementia reinforced their gendered identity as ‘doers’ and providers (Ribeiro et al., 2007).

### 3.3 Independence matters

Some participants highlighted the importance of independence for their identity and agency. Although independence is usually portrayed as a male trait or value, it was also emphasised by a minority of women (Matud et al., 2003). Hence, men *and* women echoed the devaluation of dependency inherent in society (Brannen, 2006). Maintaining personal grooming and housekeeping skills was important to these women, reflecting social expectations regarding feminine image and domestic competency. Correspondingly, the value that men placed on their freedom reflects its centrality for masculine identity and agency, but also highlights how dementia poses challenges for liberty (given the risk of institutionalisation).

Two women emphasised their independence, notably their self‐care and domestic competencies (or executional agency). Colleen was emphatic that she did not need any help with dressing—“no, no, no” (she had impaired speech). When asked who cleaned the house, she repeated in a pleased tone “me, me” and jokingly disparaged her husband's contribution: “(he) practically do [pause] none [pause] nothing”. She conveyed pride in her independence, albeit her abilities had declined from prior to the dementia. Dorothy viewed herself as “fortunate” because she could care for herself, but she also valued interdependence, as she enjoyed caring for others. Although health professionals and her husband had persuaded her to attend a day centre, Dorothy had tried it and disliked it. In refusing formal care, she again emphasised her self‐care ability: “I feel I'm coping alright … so um, I don't need to, uh, go and have people helping me”. While she asserted her agency in the face of their ‘caretaking’ assumptions, her contradictory need to help others but avoid dependency herself reflects policy ambiguity about striving for independence while disregarding interdependence. As these women appeared to *defend* their ability to care for themselves, this suggested a sense of vulnerability, given that such competencies are often subject to scrutiny following diagnosis.

Three men highlighted the importance of self‐reliance, freedom of movement and liberty for their masculinity (reflecting the liberal concept of agency as freedom). They disliked being dependent, particularly any assumption that they might need ‘care’. While they recognised that they were dependent on their wives now, the men found this hard to accept. Martin sighed when he said: “I do depend on Gail, but that's good for me, that, *I think*”. As regards a loss of freedom of movement, Martin said that getting lost had made him feel like a little boy, thus challenging his manhood: “we like to go out together. And I feel safer, because sometimes I get lost … And I felt like a little boy, ‘I want me mummy!’ … a man of six foot and I was, I was nearly crying”. Gordon spoke metaphorically about being under surveillance, whereupon he asserted his freedom via embodied agency: “somebody trod on my toes … And I packed a bag and walked out of the house … it put a stop to my freedom so I can't go out now … (because *they* say:) ‘Wait a minute, I can't let you go on your own, you need to be watched’”. Gordon's allusion to ‘they’ suggested that he was partly referring to healthcare staff: “and then they're not spying on me, they, they are looking after my welfare and, as my wife's among them, I'm sure they're doing the right thing”. However, he evidently understood that his movements were being monitored to protect his well‐being.

Liberty was a sensitive issue (explicit or implicit) for two men who had previously been admitted to care homes. Alfred only mentioned in passing that he had stayed in a care home when his wife went on holiday, seemingly to avoid any discussion: “I go in a home”. When asked how he had felt about being admitted, he simply said: “er, I just hope that Susan can have a good time”. His lack of emotion reflected his depressed state (his wife said he had been diagnosed with depression) but perhaps also a masculine unwillingness to show vulnerability (Shields et al., 2006). In contrast, Gordon clearly viewed his admission as an imprisonment: “…especially after I was imprisoned! If (when) they put me under [inaudible] imprisonment of the hotel”. His choice of language indicated that he had felt very confined by the admission, thereby undermining his masculine sense of freedom. As his comment suggested he had not made the decision about the admission himself and, indeed, Gordon's wife said (in her interview) that she had made the decision, there had been limited scope for him to exercise deliberative agency here.

### 3.4 Women caring

Four women highlighted how they cared for others (practically and emotionally), indicating the reality of interdependency even in the lives of people who are assumed to be dependent (Davies, 2011). Dorothy conveyed her pride in helping others, including visiting a friend who was unwell: “I like to uh, help people and uh, go and uh, go and uh, spend time with them”. Amanda attended a day centre but preferred to stay at home and care for her husband when he was ill: “when Henry's off‐colour, I'd rather stay and look after him”. Although she viewed herself as his carer now, she had previously been the breadwinner, necessitated by his ill‐health. Ironically, Jenny was caring more for her husband than vice versa. While Marcus was physically healthy, he was emotionally dependent on his wife. As she was quite an independent woman, some of his vulnerability arose because he perceived that she did not need him. Jenny conveyed emotional reflexivity when she discerned her husband's need for *her* psychological support: “he relies on me a lot, er, because he's, he loses confidence in himself … Er, and I do need him, I've got, got to reassure him now”. As Jenny felt that Marcus would be unable to cope if she died, but she could manage on her own if necessary, this pointed to her enduring sense of feminine agency, irrespective of her dementia: “er, he would go into a decline, I'm sure he would”. Traditionally, men were socialised into being strong and women were expected to be vulnerable and emotional, but the dynamics in this couple demonstrated the opposite, challenging such gender stereotypes (Sen & Ostlin, 2008).

### 3.5 Women's religious beliefs

Three women emphasised the role of religious beliefs in guiding their lives. When asked if her religion was important to her, Colleen said: “yes, it is”, reached for her bible and named her church (she had impaired speech). She and her husband were committed to the same Christian religion, but religious practice was not necessarily shared in other couples, which could cause tensions (mentioned earlier). Dorothy viewed her religion as a spiritual anchor: “it's part and parcel of my life you know and, uh, it's what I believe in”. As she said she would “feel lost without going to church”, it appeared that, subconsciously, Dorothy felt that her dementia would lead to her becoming disoriented. In referring to her faith as “a walking stick”, it seemed that Amanda looked to her religion to help her manage any future dependency. Ironically, she reflected at times on whether God or her husband was most important in her life and, as she usually favoured God, this indicated her spiritual sense of agency (and humour): “I debate between the Lord and Henry. Often Henry loses”. Women also cared spiritually for others—for example, Dorothy said she liked to “pray for people”. Religion clearly enhanced some women's sense of agency, particularly when faced with their condition, even if it did not translate into apparent control over their lives. Although current debate critiques a concept of agency centred on rationality, spiritual agency is usually disregarded.

## 4 Discussion

Women and men with dementia defined themselves according to their social and gender roles. As relationships were central to women's identity, they sometimes prioritised harmony within their marital relationships over their own well‐being. Indeed, women are expected to ‘sacrifice’ their own health in order to meet the needs of their families (Sen & Ostlin, 2008). In contrast, men highlighted the significance of employment for their masculinity. However, it may be that people with dementia were ‘doing gender’ in order to emphasise their enduring competencies to the researchers (West & Zimmerman, 1987). Although the participants conformed to gendered “norms”, their attitudes or behaviours also challenged gender stereotypes, such as when women disliked housework and men revealed their emotional natures. As they perceived themselves in terms of their gendered identities, this disrupted the dominant social construction centred on dementia. Similarly, a Swedish study of identity in people with dementia found that the negative impact of the condition was not a significant theme in their life stories (Westius, Kallenberg, & Norberg, 2010). Although the lack of salience of a dementia identity might be construed to demonstrate limited awareness of the condition, the data highlighted when the participants referred to their condition, often implicitly. Although disability is generally assumed to be central to self‐concept (Fine & Asch, 1988), on the contrary, women and men resist the imposition of a negative, dementia identity and seek recognition for their established social personas. In contrast to the focus on personal identity in the literature, it is clear that people living with the condition view themselves intersubjectively. More specifically, they retain a perceptive understanding of the dynamics of intimate relationships and endeavour to remain involved in family and community life.

As work and freedom were central to men's agency, this pointed to an instrumental and singular notion of agency, whereas relationality and care informed women's agency. Similarly, previous authors have highlighted that men's reflexivity is often centred on self‐fulfilment while women prioritise the needs of others (Britton & Baxter, 1999; Hoggett, 2001). However, as women and men equated agency with independence, this suggested a more individualised concept. Although their references to independence focused on physical self‐reliance, they also alluded to a potential loss of cognitive independence. Their association of agency with independence reflects dominant discourse which has paradoxically excluded or devalued disabled people (Bakhtin, 1994; Reader, 2007). Equating agency with independence ignores how disabled people can exercise agency, but may need support to do so. Indeed, as current theory is dominated by an individualistic and “strongly purposive” concept of agency, this has contributed to interdependency being devalued and to people with dementia being perceived to lack agency (Emirbayer & Mische, 1998, p. 984). Yet, it is important to recognise that cognitively disabled people can still exercise agency.

Although care influenced feminine agency, of course, women are often constrained by societal expectations regarding their caring responsibilities (Finch & Mason, 1993). Similarly, the dialogue pointed to tensions within some couples associated with gendered expectations, for example, regarding housework. The priority women gave to their marital relationships showed their relationality, and also suggested a degree of gendered acquiescence. Nonetheless, they also resisted gendered assumptions regarding their marital responsibilities. Correspondingly, as ‘being in charge’ informed men's agency, tensions arose when a minority of men sought to exercise decision‐making authority but this was challenged by the condition. Men's dependency on their wives could also undermine their sense of masculinity. Thus, gender dynamics influence the exercise of agency even in dementia.

The literature had suggested that agency might be a gendered concept and, indeed, it appeared that men were somewhat individualistic and rational in their concerns, whereas women were more relational and even spiritual. However, women *and* men showed their socio‐emotional reflexivity and, at times, it appeared that men were more emotive than women. Yet, the masculine model of reflexive agency disregards how men's reasoning is emotionally informed (Hoggett, 2001). The dialogue of women and men revealed their own voices and echoed the voices of tangible others (e.g. family, professionals and ex‐colleagues) and a “generalised other” in terms of societal expectations (Mead, 1934). Thus, dialogical analysis can illuminate the internal and interpersonal devices that women and men with dementia use to manage their presentation of self when negotiating social relationships and the subtle exchanges which manifest gender relations and even inequality (Goffman, 1990).

## 5 Conclusions

The ‘ungendering’ of people with dementia in policy discourse is part of the social process of disability by which established identities are often rendered invisible and a dementia identity is imposed. In addition, as cognitive psychology research on dementia has focused on individual identity, this has contributed to the neglect of the social self. Yet, women and men clearly perceive themselves via their gendered identities, such that a critical perspective is needed in dementia policy and practice whereby their authored identities are privileged. Although the WHO highlighted the need to take account of gender when improving population health, it has not viewed gender as similarly important for well‐being in dementia (World Health Organization, 2012, 2013). Yet, health and social care staff should recognise and facilitate the gender identity and related social roles of people with dementia (e.g. parent, carer and worker) in order to enhance their quality of life.

Viewing reflexivity as a socio‐emotional (rather than a cognitive) process enables the agency of cognitively disabled people to be more readily recognised. It is noteworthy that agency can take gendered forms irrespective of dementia, but also that emotion informs reflexivity even in men. As agency theory has lacked an empirical basis and dialogical methodology has been narrowly focused on internal conflict (Burkitt, 2010), future research should link dialogue with the structures and practice of social (in)equality in order to inform debate about gender and health. In contrast to the over‐reliance on language in agency debate, it was notable that people with dementia often expressed their agency in embodied and emotional forms, including via humour. Thus, broadening our understanding of what constitutes communication and ‘dialogue’ would help to facilitate the gendered voices of people living with this condition and promote their relational agency.

## Conflicts of interest

There are no conflicts of interest to declare.

## Endnote

1 Pseudonyms are used (first names where preferred by participants).
